# Improving public health evaluation: a qualitative investigation of practitioners' needs

**DOI:** 10.1186/s12889-018-5075-8

**Published:** 2018-01-30

**Authors:** Sarah Denford, Rajalakshmi Lakshman, Margaret Callaghan, Charles Abraham

**Affiliations:** 1 Children’s Health and Exercise Research Centre, Baring Court, St Lukes Campus, University of Exeter, Exeter, EX1 2LU UK; 2 University of Cambridge, Cambridge, UK; 3 Institute of Health Research, University of Exeter Medical School, Exeter, UK

**Keywords:** Public health practitioners, Public health, Evaluation, Qualitative, Interviews

## Abstract

**Background:**

In 2011, the House of Lords published a report on Behaviour Change, in which they report that “a lot more could, and should, be done to improve the evaluation of interventions.” This study aimed to undertake a needs assessment of what kind of evaluation training and materials would be of most use to UK public health practitioners by conducting interviews with practitioners about everyday evaluation practice and needed guidance and materials.

**Methods:**

Semi-structured interviews were conducted with 32 public health practitioners in two UK regions, Cambridgeshire and the South West. Participants included directors of public health, consultants in public health, health improvement advisors, public health intelligence, and public health research officers. A topic guide included questions designed to explore participants existing evaluation practice and their needs for further training and guidance. Data were analysed using thematic analyses.

**Results:**

Practitioners highlighted the need for evaluation to defend the effectiveness of existing programs and protect funding provisions. However, practitioners often lacked training in evaluation, and felt unqualified to perform such a task. The majority of practitioners did not use, or were not aware of many existing evaluation guidance documents. They wanted quality-assured, practical guidance that relate to the real world settings in which they operate. Practitioners also mentioned the need for better links and support from academics in public health.

**Conclusion:**

Whilst numerous guidance documents supporting public health evaluation exist, these documents are currently underused by practitioners – either because they are not considered useful, or because practitioners are not aware of them. Integrating existing guides into a catalogue of guidance documents, and developing a new-quality assured, practical and useful document may support the evaluation of public health programs. This in turn has the potential to identify those programs that are effective; thus improving public health and reducing financial waste.

## Background

Public health interventions have the potential to improve population health [1] but often lack convincing evidence bases [2]. It is strongly recommended that a program be systematically developed and evaluated [3, 4]. Without rigorous evaluation, it remains unclear if interventions are effective (i.e., responsible for any observed changes in health), for whom they are effective, when they are effective (i.e., social and contextual factors that may impact on the effectiveness of the program), which processes or mechanisms explain effectiveness and whether or not the intervention generated adverse or negative effects [5]. Indeed, in 2011, the House of Lords published a report on Behaviour Change, in which they report that “a lot more could, and should, be done to improve the evaluation of interventions” [6]. Quality evaluation is essential to the development of an evidence base for the effective population-relevant interventions that can be integrated into public health policy and practice [5]. Yet evaluation of complex public health interventions is challenging and there is a need for guidance as to how best to evaluate.

Evaluation guidance is available. For example, in 2010, the UK Medical Research Council published a guide to support researchers who are developing, implementing or evaluating complex interventions [3]. The UK National Obesity Observatory has developed Standard Evaluation Frameworks (SEFs) for interventions programs targeting obesity, healthy eating and exercise [7]. These SEFs are written for practitioners, and provide useful checklists of essential and desirable data to be collected as part of an evaluation. The World Health Organisation (WHO) have developed guidance documents relevant to evaluation; including evaluation of health promotion evaluation, economic evaluation, and evaluation of community based interventions [8]. The United States Centre for Disease Control and Prevention (CDC) developed a framework aiming to provide a systematic way to approach evaluation using a series of steps and standards [9]. Accompanying guides were also developed; including both topic specific and generic guides on various aspects of evaluation (e.g., planning evaluation, process evaluation, economic evaluation). HM Treasury produced the Magenta Handbook – specifying recommendations of what to consider when evaluating an intervention; and the related Green Book of recommendations in relation to conducting an economic evaluation [10]. The website “Better Evaluation” was founded by several international partners, and provides step to step guidance on planning and managing evaluation and monitoring processes and links to multiple resources [11].

Many more guides to evaluation are available; a recent search identified a total of 402 documents that can be used to support the evaluation of public health programs. This large literature is not integrated, easily accessible or graded in terms of quality assurance and there is no guide to navigating existing resources or selecting guidance appropriate to particular projects. The literature is comprehensive; including information on both topic specific and generic evaluation guidance, ranging from very basic to very detailed information on multiple facets s of evaluation, and targets a range of expertise from novice to experienced evaluation practitioners. This makes it overwhelming, and difficult for public health practitioners to navigate the literature and identify the guidance document that is suitable for their needs and level of expertise.

So what do public health practitioners need to better understand and apply evaluation guidance? In the present study we aimed to assess the needs of public health (PH) practitioners in relation to evaluation guidance materials and training. We sought to understand which guides are used and which are not, why guides are used or not, what is lacking from current guidance documents, and what can be done to better support the conduct of public health evaluation. We anticipated that our findings would contribute to the development of useful and practical guidance to evaluation.

## Methods

### Design

Qualitative interviews were conducted with public health practitioners in two UK regions. The interviews focused on existing evaluation practice and their needs for further evaluation guidance training.

### Sampling and participants

Participants were selected from two regions in England. The two regions were selected for pragmatic reasons as the authors have contacts with public health practitioners in these areas. The areas are similar in population size, ethnic diversity, life expectancy and deprivation. We invited PH practitioners in the two regions via email invitations to:

1. Directors of PH and leaders of PH teams in each area.
2. Other senior PH practitioners known to the research team
3. PH participants who attended an evaluation workshop in one of the two areas.

Potential participants were invited to be interviewed at a time and place to suit them, and asked to recommend any other practitioners who might be interested to participate. Prior to taking part in the interview, participants were provided with an information sheet, informing them of the nature and purpose of the study, and stating that they could withdraw at any time. All participants signed a consent form to say that they were willing to take part in the interview, that they were willing for the interview to be recorded, and were happy for select quotes to be published. They were assured that data would be anonymised and stored on password protected files, and that no one apart from the researchers responsible for the analysis would be able to link statements to them.

Thirty two practitioners were interviewed across two UK regions by the first and second authors. The first author is a health psychologist with expertise in qualitative interviews and data analysis. The second author is public health practitioner with expertise in public health research.

### Interviews

Materials for the study were approved by a local ethical committee (University of Exeter Medical School Ethics Committee). These included an interview topic guide that included questions on (1) current PH evaluation training, (2) current public health evaluation practice, (3) materials and procedures used in current practice, (4) desirable evaluation training at different levels, (5) needed resources and guidance materials that would be relevant to, and easily-used in, public health everyday practice. The semi-structured interview schedule consisted of a series of questions pertaining to these five areas. Prompts were used to elicit further detail when necessary.

### Data analysis

Interviews were audio recorded, anonymised and transcribed verbatim. We analysed the data using a thematic approach [12]. Three researchers independently read transcripts and noted down core codes that were identified. Through discussions, a list of preliminary themes was developed [13]. As analysis progressed, the existing list was refined, and related themes grouped together [13].

Interview data that was related to each theme was copied and pasted into relevant tables. We used these tables to identify narratives within cases and diversity between cases. Divergent cases were discussed and included in the thematic analysis [14].

## Results

### Participant details

Interviews were conducted with a total of 32 participants; including directors of public health, consultants in public health, health improvement advisors, public health intelligence, and public health research officers. Interviews lasted between 15 and 32 min (mean 27 min).

### Results of the analysis

Four overarching themes were identified and representative quotes were selected. The themes were: (i) the need for evaluation (ii) training in evaluation (iii) evaluation guides and resources (iv) external support.

### The need for evaluation

Respondents expressed a need for and commitment to evaluation. They were also clear that when evidence of effectiveness is strong, further evaluation is not needed, and it was suggested that time and resources should be devoted to evaluating the effectiveness of less well-evidenced interventions.

*“I think what we do need to evaluate, and to get a better grip of, is some of our unknown territory in public health” (participant 4)*

Moreover, with limited funds available, cost and cost effectiveness were regarded as crucial. Several interviewees highlighted the importance of accountability, effectiveness and efficiency when using public money.

*“I think any new projects that involve large amounts of money or investment, and amounts of peoples’ working time, they should ideally be evaluated to see if they’re worthwhile rather than just being repeated on a yearly basis without ever really knowing if they’re effective or not” (participant 6)*

Practitioners were clear about the need to justify costs and choose one intervention over another and, given this, the need for evaluation of interventions. This was a recurring theme across interviews, highlighting practitioners’ motivation to demonstrate that their programs are effective, worthy of continued funding, and that public money is spent in the best way to meet the needs of the population.

*“I’ve got a given budget and a given amount of money, so how do I best spend that for my population?” (participant 9)*

### Training in evaluation

Most practitioners had received some training in evaluation but to varying levels, resulting in clear differences in confidence in being able to undertake evaluations. Many had completed Masters in Public Health and some had completed other post graduate degrees. Some had little formal training but, nonetheless, had learned evaluation skills through working with more experienced evaluators.

*“I don’t think I’ve ever had any formal training on evaluation. But what I have done is learn on the role and I’ve been I’ve looked for examples of best practice and stuff elsewhere” (participant 14)*

Many felt the need for further training in specific areas of evaluation (i.e., economic evaluation), and/or refresher courses. This was particularly true for those who did not do evaluations on a regular basis.

*“I think always [a need for training] to keep your skills up… if you have been on evaluation training that’s great - but I think it’s always useful to have a sort of refresher and, you know, there might be new things coming about” (participant 1)*

In addition to training in how to do evaluation, it was felt that there was a need for training in how to critically appraise evaluation; as this is what practitioners are likely to have to do on a regular basis.

*“I think the training that any public health person needs is to be able to spell out the parameters very clearly rather than being able to do it. You know I have training in critical evaluation for example and that’s a skill you are more likely to use more regularly than evaluation” (participant 7)*

### Evaluation guides and resources

Apart from the Public Health England Standard Evaluation Frameworks (SEFs), evaluation guidance documents were rarely used. The main reasons reported for not using guides included: 1) not being aware that relevant guides exist or being unable to find them 2) too many guides with no rational for choosing between them 3) no useful or practical guides that are fit for purpose. However, SEFs were considered very useful and practitioners suggested that similar frameworks should be developed for areas as yet not covered by SEFs including sexual health, tobacco use control, mental health promotion, falls prevention and workplace health promotion should be developed. Participants discussed the need for one quality assured, nationally recognised document that would be considered the gold standard for evaluation of public health interventions. Other participants felt that the development of additional guides was futile, and felt that a guidance document, in which the existing, disparate body of literature is summarised, and relevant documents signposted would be preferable. They suggest ways in which such documents could be made developed, and what they should contain.

Despite the abundance of evaluation guides, practitioners reported that they were unaware of them, could not find them, or did not find them to be fit for purpose.

*“Most of the time, I’ll be honest with you, it’s searching online, it’s looking at the relevant websites… and taking elements of various evaluations and designing them myself” (participant 19)*

These practitioners suggested that there was a need for one gold standard, overarching guide to evaluation. Such a guide – or guidance website - should be an authoritative national guide on how to conduct a meaningful evaluation, be practical, succinct, user-friendly and available online. It should also include examples/case studies of good evaluations and should serve as a repository for future evaluations.

*“I think if there was national guidance on what was expected in an evaluation, that might be useful, then everybody is working to the same document. Because I think there’s lots of local things floating around that only a few people have access to, so having a national one there everybody in public health could access” (participant 8)*

Practitioners stated that such a guide should include advice on ‘optimum’ (gold standard) and ‘practical’ (good enough) designs and data collection, and should also include examples/case studies of good evaluations and should serve as a ‘searchable repository’ for future evaluations. However, as noted above, the key motivation underpinning evaluation is to identify interventions that are effective and good value for money. Consequently, evaluation in relation to the national public health outcomes framework was frequently called for.

*“It has to be related to outcomes, and it has to have an economic dimension to it. And then you know…I think that’s absolutely critical because that’s all I get asked for nowadays is economic stuff” (participant 3)*

Such a guide should be generic, adaptable to meet the wide variety of needs that exist, and should highlight the need for pragmatic approaches rather than focusing on what would be considered high quality by academic standards.

*“It needs to come from that kind of pragmatic approach of what’s good enough in a service setting, not just sort of scare people off with the…you know, this is the pure academic approach to doing an evaluation, and if you can’t do it like this it’s not worth doing it at all” (participant 7)*

Crucially, it was noted that any guidance document should be developed jointly between practitioners and academics.

*“I think it’s one thing producing a resource, but it’s the challenge is getting it useable for the audience that you’re aiming it at, and you can’t…I mean there’s no hope of getting it used if it’s not going hit the right audience” (participant 5)*

Of the practitioners who were aware of the volume of resources that currently exist, many stated that they were lacking the time and the knowledge to explore these in any detail, to find those that are of high quality, relevant to their evaluation needs, and suitable for their level of expertise. It was felt that development of an additional guide to evaluation would be unhelpful – given the quantity that already exist. Instead, practitioners suggested that existing guides could be presented in one place with signposts to relevant guides, advice on how and when to use each guide, and an assessment of the quality of each guide.

*“Something that probably is quite good would be to almost have these [guidance documents] in one place and almost like a…a web link kind of thing, you know, what type of evaluation are you interested in, oh we think this resource would be good for you. That could be quite useful” (participant 16)*

### External support

Many interviewees also called for better practitioner/academic links. Practitioners wanted help planning evaluations, managing projects, and answers to specific questions should they arise. However it was recognised that it would be difficult to provide a comprehensive service without more resources. Some interviewees suggested academics taking a more commercial approach and either being funded to provide this support or acting as consultants.

*“I don’t think it’s realistic to support everyone everywhere out there, but actually some guidance more, you know, frequently asked questions and things… I suppose some sort of helpline… so people go to the frequently asked questions, do a bit of work beforehand and then someone to speak to and say ‘would this work if I did this, or will it give me anything useful or will it be invalid or whatever or reliable or something” (participant 18)*

Practitioners wanted someone to scrutinise their evaluation plans and provide advice rather than carry out the evaluation so that they (practitioners) could develop their skills over time and the evaluations would be fit-for-purpose.

However, whilst some practitioners felt that evaluations carried out by researchers did not meet the needs of practitioners themselves, others felt that all evaluations should be conducted by academics. These practitioners were adamant that practitioners did not have the skills or resources to complete evaluations themselves. Instead, felt that there was a need to learn how to frame questions appropriately and sufficiently - so that they may then be passed to academic partners, who could complete the evaluation on their behalf:

*“You can develop another sort of another guidance tool the question is who is going to use that guidance… I am giving you the responses from the perspective of a public health expert who works in local government. It’s a very, very different environment and what you need, what the public health experts in local government needs desperately is the ability or the toolkit to be able to frame a question correctly - not to be able to answer it. Because they don’t have the resource to answer it, they don’t have the capability to answer it and it will not necessarily be the best value for money for them to be the ones necessarily answering it” (participant 7)*

However, there was a feeling among most interviewees that staff involved in evaluation at different levels should understand how it applied to their roles; for example data collection would be more effective if the collectors understood how the data lead into change and commissioners and politicians would be more likely to fund evaluation if they could see how it worked. For example:

*“Even if you commission it you still have to know what you’re commissioning, you know, you still have to know what’s expected from a qualitative survey, what’s expected from a quantitative survey and so on and so forth” (participant 24)*

## Discussion

Public health practitioners need to justify expenditure on interventions and programmes to ensure their initiation or continuation. This requires evidence of benefit which, in turn, requires evaluation. We interviewed 32 practitioners’ across two UK regions to explore their views on evaluation and what guidance and help they needed to embed evaluation into everyday practice.

Practitioners were clear that not all practice needs to be formally evaluated, especially that previously shown to work and, of course, not all practice can or should be evaluated [15]. Nonetheless, it is worth noting that interventions that are “known to work” may not be effective if implementation varies across context and, moreover, even when implemented with fidelity is achieved interventions may not work or may work differently when contextual variation impacts on mechanisms of action. So what is known to work may be less clear if transferability and replicability are complex [5].

Whilst numerous evaluation guides and guidance documents exist [3, 5, 7, 8, 10, 11] these are rarely used by practitioners – either because they are not aware of them, or because they are not considered to be useful. This may in part, be due to the fact that they were not developed with public health practitioners or developed by a leading organisation. Indeed our research shows that the resources considered most useful were the standard evaluation frameworks developed by Public Health England. These frameworks were developed by a nationally recognised and highly regarded organisation, included considerable collaboration with public health practitioners, and the final document is widely promoted. This means that the content of the guide is relevant, that practitioners are aware of the guide, and have confidence in the recommendations provided.

Many practitioners had received training in evaluation but felt that they were still lacking the necessary skills to conduct evaluations without further training or support. This concurs with previous research, which identified a number of factors that affect practitioners’ abilities to evaluate programs [16]. Lobo and colleagues discuss how practitioners are limited by a lack of knowledge and skills relating to the evaluation of programs, and lack the skills to apply evaluation frameworks in a useful way [16]. They suggest strategies to overcome this; including access to case studies, mentoring and additional training in evaluation. This concurs with our participants’ suggestions that additional guidance should include mentor support, case studies and additional training.

Practitioners in the current study wanted greater access to researchers with expertise in evaluation. They acknowledged that while they needed to enhance their own evaluation skills, there would still be a need for consultation and collaboration with expert evaluators. However, whilst this is the ideal, there are problems making this a reality. Previous research has identified “disconnections” between practitioners and academics; relating to the way in which problems are identified and addressed [17]. Such fundamental differences in approaches to evaluation in terms of timing, resources, use of theory, and focus on internal versus external validity make partnerships problematic. Awareness of discussions about these issues at the onset of collaborations are essential – and will ensure solutions can be negotiated at the onset. Participants in the current study spoke about the need for advice to be practical, and not overly academic. Stakeholder involvement in the design and development of any partnership, guidance document, or resource is crucial.

Within the current study, practitioners suggested that in an ideal world, guidance would need to be both specific, yet generic, focusing on the optimal gold standard, but also highlighting the practical (good enough) approach. It would necessarily be accessible for the novice, but detailed enough to be useful. However, practitioners were of the opinion that this just isn’t possible or desirable in one guide, and discussed the need for existing literature to be made more accessible. Participants discussed a guide to the large body of guidance; that would pull the existing literature into one place, and act as a sign post to support practitioners identify the relevant documents for their needs, as well as quality assurance. Researchers funded by the National Institute for Health Research (NIHR) School for Public Health Research (SPHR) have developed such a document in collaboration with public health practitioners [18]; however, its utility is yet to be established.

## Limitations

Our study was based on sample of public health practitioners from two regions in England. This may limit the transferability of the findings; although the regions were large and spanned a number of diverse towns and cities. Further research is needed to explore the views of practitioners in other regions with varying levels of deprivation. Furthermore, our sample were self-selected; thus indicating an interest in the topic of evaluation. However, our participants had a range of opinions and practices on evaluation. This lack of consistency in the answers provided highlights the range of practices that are currently occurring within public health departments – even amongst those largely positive about evaluation.

## Conclusions

Whilst numerous guidance documents supporting public health evaluation exist, these documents are currently underused by practitioners who are unaware of them or do not consider them useful. Practitioners wanted 1) improved training in public health evaluation, 2) an authoritative, national guide on evaluation relevant to national policy priorities, 3) a website in which existing guides and evaluations were presented, reviewed and quality assured, and 4) improved links between themselves and researchers with evaluation skills. Providing these supports could raise standards of evaluation in public health practice and so clarify which interventions and programmes are worth continuing or initiating.
